# Hierarchical Interfaces as Fracture Propagation Traps in Natural Layered Composites

**DOI:** 10.3390/ma14226855

**Published:** 2021-11-13

**Authors:** Hanoch Daniel Wagner

**Affiliations:** Weizmann Institute of Science, Rehovot 7628604, Israel; daniel.wagner@weizmann.ac.il

**Keywords:** interfaces, fracture arrest, biological composites, layered structures, crack deflection

## Abstract

Compared with their monolithic version, layered structures are known to be beneficial in the design of materials, especially ceramics, providing enhanced fracture toughness, mechanical strength, and overall reliability. This was proposed in recent decades and extensively studied in the engineering literature. The source of the property enhancement is the ability of layered structures to deflect and often arrest propagating cracks along internal interfaces between layers. Similar crack-stopping abilities are found in nature for a broad range of fibrillary layered biological structures. Such abilities are largely governed by complex architectural design solutions and geometries, which all appear to involve the presence of various types of internal interfaces at different structural levels. The simultaneous occurrence at several scales of different types of interfaces, designated here as hierarchical interfaces, within judiciously designed layered composite materials, is a powerful approach that constrains cracks to bifurcate and stop. This is concisely described here using selected biological examples, potentially serving as inspiration for alternative designs of engineering composites.

## 1. Introduction

The issue considered in the present communication, in a mostly observational and qualitative way, deals with selected sophisticated design solutions offered by specific natural architectures to the problem of blocking fracture propagation in layered composite structures. The interest in this problem, which has been extensively examined over the last few decades in the literature, originated in the brittle nature of engineering ceramic materials under tension or bending, despite their otherwise excellent thermomechanical properties. Indeed, modern structural materials are often used in critical applications such as aircraft jet engines, where reliability is the key property, and thus sudden, unstoppable fracture is unacceptable. For those structures, toughness is at least as important as strength (if not more important). It is only recently that physics- and materials-based research has concentrated on the quest for materials and/or structures possessing high simultaneous strength and toughness [1]. Perhaps surprisingly, such structures are frequently found in nature, which explains the recent interest in studying them. Here, a brief summary is given of the mechanical function of interfaces in synthetic (engineering) layered materials and structures. This is followed by a description of less well-known design patterns produced by nature to generate crack bifurcation and arrest in complex layered structures.

## 2. Interfaces in Engineering Materials

**Figure 1:**
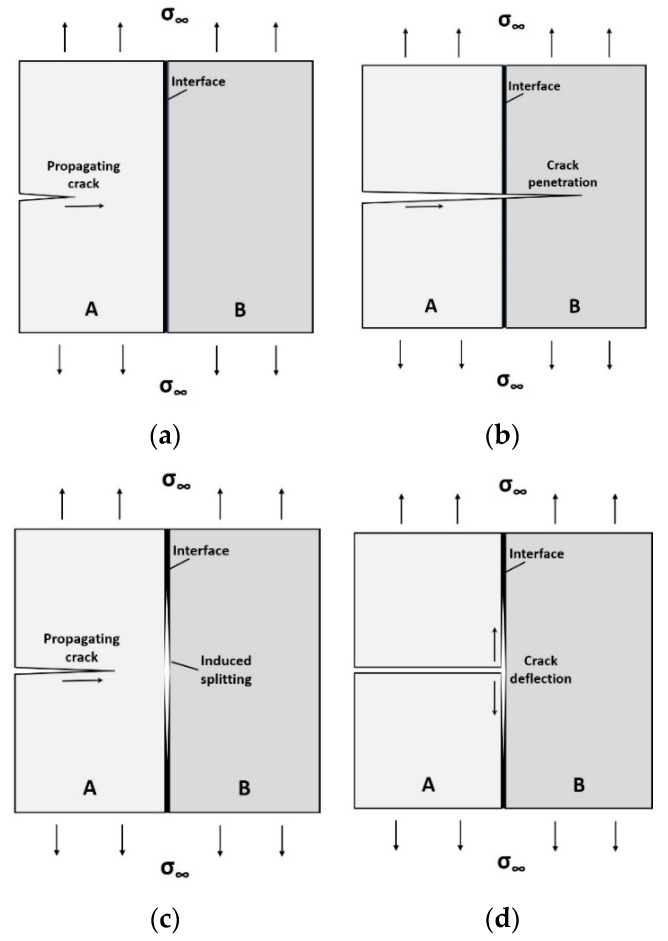
Propagation paths of a crack in a bi-material layered structure: (**a**) initial state; (**b**) crack crossing an interface in a self-similar fashion; (**c**) crack splitting at interface ahead of the crack tip (the Cook–Gordon mechanism [2]); (**d**) crack bifurcation at interface.

The brittle rupture of monolithic engineering materials may be overcome by using layered architectures, a technique that simultaneously enhances the energy needed to fracture the structure and its overall reliability. Let us consider a simple, two-layer structure, as in Figure 1. When a sharp crack propagates toward the interface, Figure 1a, several scenarios may arise. If a relatively strong interface is present, the crack will run through material A, cross the interface and penetrate into material B with no change in direction, Figure 1b, leading to rapid (brittle) failure. By contrast, a weaker interface may split well before the crack tip reaches the interface (this is the well-known Cook–Gordon mechanism [2]) as in Figure 1c, or, alternatively, cause the propagating crack to deflect relative to its original (self-similar) direction as soon as it hits the interface, as in Figure 1d. Both bifurcation mechanisms at a bi-material interface not only delay the fracture process (and thus increase structural reliability), but are also a source of increased toughness through additional mechanisms, such as the pulling-out of ligament bridges from the matrix [3].

The propagation of cracks along interfaces makes it possible to hamper the evolution of damage by complicating the fracture paths. Since rapid failure is delayed or avoided, structural reliability is increased even if materials A and B are fragile on both sides of the interface. Whether a crack tends to propagate parallel to itself in a Griffith-like fashion or bifurcate in a deflected direction (not necessarily perpendicular to its original path) has been thoroughly studied for the bi-layer case [4,5,6,7,8]. The elegant model of Kendall [4] can, in principle, be generalized for a multilayer–multimaterial configuration [9].

Based on the above, practical ways of preparing tougher ceramics and ceramic-based composites by introducing weak interfaces that deflect growing cracks have been proposed by Evans [10] (among others). Clegg et al. [11] described a simple, inexpensive way of preparing a ceramic material that contains such interfaces. Silicon carbide powder was made into thin sheets, coated with graphite to provide weak interfaces, pressed together, and sintered without pressure. Relative to the monolithic material, the apparent fracture toughness for cracks propagating normal to weak interfaces increased more than fourfold, and the work required to break the samples increased by a factor of more than a hundredfold. Mayer [12] noted the controlling role of special architectures and thin viscoelastic organic layers in the energy dissipation in these structures. In our recent work, composites were prepared from high-grade commercial alumina with spin-coated interlayers of ductile polymers (PMMA and PVA) [13]. In some cases, the fracture toughness of the composites was increased by up to an order of magnitude. In another work, evaporation-driven self-assembly (EDSA) was used to deposit a thin network of multi-wall carbon nanotubes on ceramic surfaces, thereby generating an interphase-reinforcing layer in a multiscale laminated ceramic composite. Both strength and toughness were improved by up to 90% while keeping the overall volume fraction of carbon nanotubes in a composite below 0.012%, making it a most effective toughening and reinforcement technique [14].

Similar toughening principles have existed in nature for a long time, in even more sophisticated ways, as exemplified in the next section. From these, further advances could potentially be made in future synthetic structures.

## 3. Interfaces in Natural Materials

Recent reviews focus on the relative ubiquity and mechanical significance of layered structures and interfaces in natural structures [3,15,16]. It is accepted that the deformation and toughness of natural materials are largely governed by the interfaces that join these building blocks [15,16,17,18]. These interfaces channel nonlinear deformations and deflect cracks into configurations in which propagation is more difficult. However, our quantitative appreciation of the role of these interfaces is still limited, and there are associated controversies in our understanding of how they are constructed and how they operate. Barthelat et al. [16] show that the strength and toughness of interfaces in natural materials, such as nacre, cortical bone and wood, are from two to three orders of magnitude lower than the strength and toughness of the materials themselves. As a general (apparently universal) rule, the interfaces must be (i) sufficiently strong to maintain cohesion between the building blocks and to ensure the structural integrity of the material, and, at the same time, (ii) considerably weaker than the rest of the material to channel deformations and cracks, and for the intricate architectures to generate attractive mechanisms and properties.

**Figure 2:**
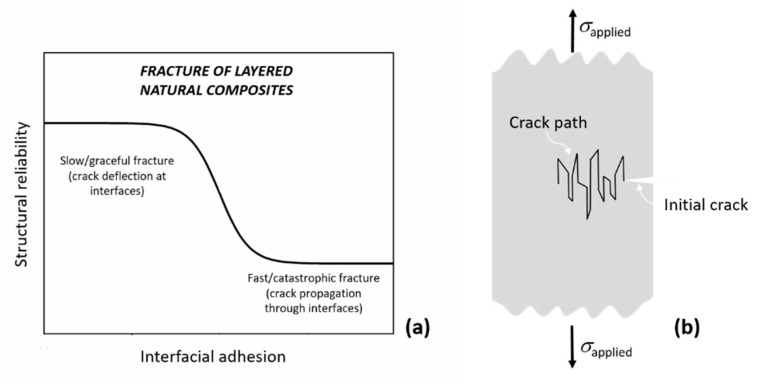
(**a**) Reliability of a layered structure as a function of the level of interfacial adhesion. Cracks do not deflect or stop at strong interfaces (low structural reliability), whereas they bifurcate or even arrest at weak interfaces (high structural reliability), as schematically shown in (**b**).

Rigid biological materials are packed with relatively softer interfaces, which can glide and slide, and facilitate crack bifurcation, thereby hindering or arresting fracture propagation. In other words, interfaces generate powerful toughening mechanisms and increase structural reliability; see Figure 2. In a material such as bone, these principles simultaneously apply over several hierarchical length scales [19,20]. Recent material models seek to incorporate the mechanical behavior of these interfaces explicitly [21], and the development of bio-inspired materials increasingly concentrates on duplicating the behavior of natural interfaces [12,22,23].

However, in contrast with the bi-material configurations discussed earlier, multi-material layered architectures, which frequently appear in biological layered structures, are much more complex to analyze and model. This is because the preferred propagation of cracks along many weaker/softer interfaces highly complicates the cracking paths. Such a toughening strategy is widely observed in biological materials, for example, in wood, bone, fish scales, plants, sponge spicules, etc. In the following sub-sections, we present and discuss the presumed effects of less well-known architectural parameters appearing in natural layered structures that should be further explored and possibly exploited.

### 3.1. Hierarchical Interfaces

**Figure 3:**
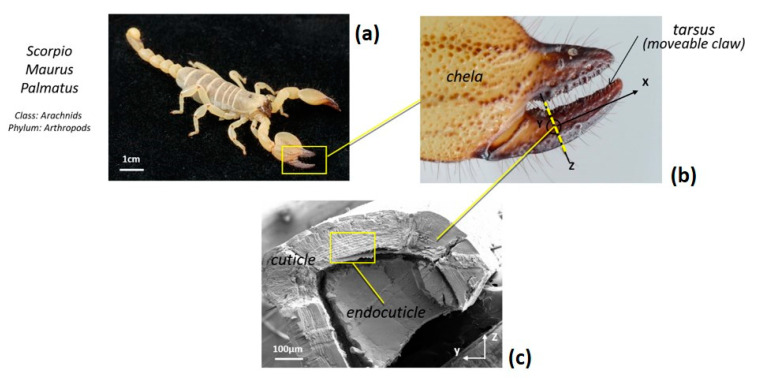
(**a**) General view of the Scorpio maurus palmatus (SP); (**b**) Detail of the tibia (chela); (**c**) SEM view of the cross-section revealing the layered structure of the endocuticle.

**Figure 4:**
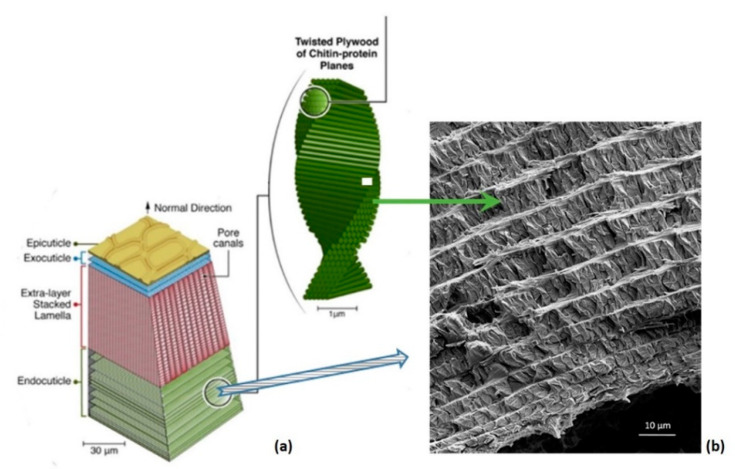
(**a**) Schematic view of the scorpion layered endocuticle and Bouligand structure; (**b**) SEM overview of the layered architecture. Each layer consists of tightly nested Bouligand laminate units (BLU).

**Figure 5:**
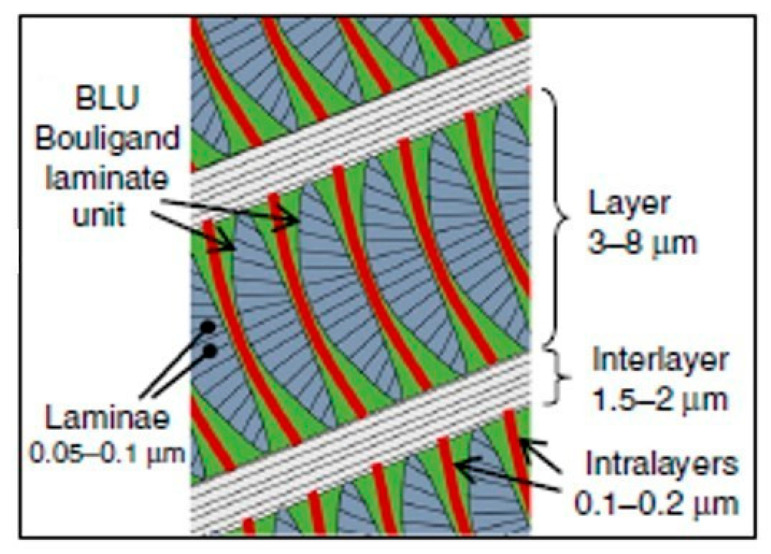
Schematic view of the scorpion layered endocuticle and nested BLUs, with definitions and scaling.

Recently, we investigated the microstructural features of the Scorpio maurus palmatus (SP) [24,25,26], which is not a dangerous scorpion to humans. Referring to Figure 3, structural analysis of the SP tibia cuticle revealed the existence of a layer made of stacked lamellae reinforced by chitin fibers, a layer that is absent in the cuticle of other arthropods. High-resolution scanning and transmission electron microscopy (SEM and TEM, respectively) and atomic force microscopy (AFM) images of the tibia cuticle revealed the microstructural details of the biological tissue. We found an unusual Bouligand architecture with varying chitin-protein fiber orientations, including the in-plane twisting of laminae around their corners rather than through their centers, and a second orthogonal rotation angle that gradually tilts the laminae out-of-plane [24,25,26]. The resulting Bouligand laminate unit (BLU) is highly warped, such that neighboring BLUs are intricately intertwined, tightly nested and mechanically interlocked (Figure 4 and Figure 5). A single BLU consists of about 40–100 laminae of chitin-protein fibers embedded in a proteinaceous matrix. Bouligand or helicoid structures are often found in living organisms [27]. First observed in 1965 by French biologist Yves Bouligand [28], they consist of layered arrangements of unidirectional fibril-based proteinaceous lamellae that follow a twisted plywood structure where each layer is rotated by a small angle with respect to the directly adjacent layer. Often based on chitin nanofibrils, such helicoids exist in the exoskeleton of arthropods such as lobsters, crabs, mantis shrimp and insects. A similar structure, based on collagen nanofibrils, is found in fish scales (in the arapaima, the coelacanth, the carp), and forms the cellulose crystallite-based walls of several kinds of plants (green algae, ferns). The helicoid motif appears to enhance the deformation and toughness of the organisms, likely through crack deflection, and to improve in-plane isotropy, as helically stacked, highly aligned layers can better withstand multi-directional forces [29].

**Figure 6:**
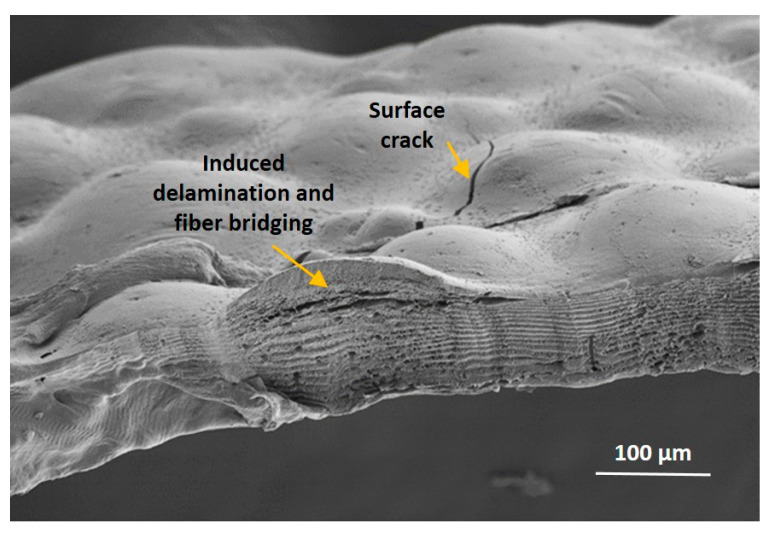
Cracks develop under load on the external surface of the scorpion tibia, which induce multiple interlayer delaminations at various depths in the endocuticle, likely through the Cook–Gordon mechanism [2]; see Figure 1c.

Under stress, the surface cracks that occur in the tibia of the SP appear to induce Cook–Gordon-like delamination patterns, as seen in Figure 6, arising from stresses just beyond the tip of the surface crack, which tend to open a perpendicular interfacial rupture (delamination). The relative intensities of these stresses were found to be such that crack deflection requires an interfacial strength five times lower than the cohesive strength [2,4].

**Figure 7:**
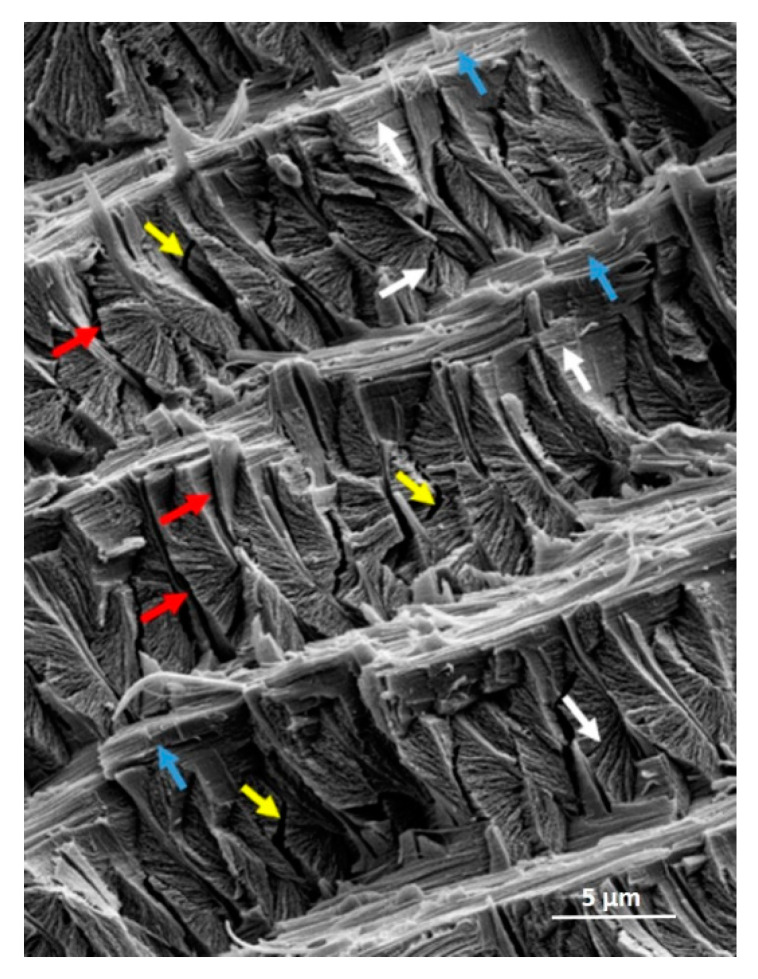
Scanning electron microscope imaging of the scorpion layered endocuticle and Bouligand laminate units (BLU) layers. White, red and blue arrows point to different interfacial failure types (refer also to Figure 5), termed hierarchical interface failures: **White**, interlamellar (nanoscale) within BLUs; **Red**, intralayer (microscale) between BLUs; **Blue**, interlayer (microscale) between layers; **Yellow** arrows designate non-interfacial (Griffith) internal cracks within BLUs.

The different types of propagating and interfacial cracks induced in the endocuticle architecture by a propagating crack under stress at different scales are most interesting (Figure 7). Such hierarchical interfacial cracks likely play the role of traps or sinks that slow down and arrest crack propagation, thereby maximizing the reliability of the tibia structure (as in Figure 2a).

The development and propagation of these hierarchical microfailures represent a potential source of energy dissipation and stress relaxation that ultimately contributes to significant damage tolerance and, thus, to structural reliability [30]. In particular, the layers of nested BLUs are capable of developing a fair amount of local relative twisting microcracks between BLUs that do not lead to catastrophic failure: the hierarchical structure of the endocuticle, with its various types of interfaces at different scales, seems to serve as a way for cracks to deflect and twist. This has important consequences, including an increased surface crack area, nested crack growth and local (and thus global) toughening as the applied force necessary to further damage the microcracked structure must constantly increase, generating further resistance in the material. It is interesting to note that BLUs may appear to act as fracture energy traps/sinks by absorbing fracture energy from the multilayer structure and dissipating it, thereby contributing to crack arrest. In a sense, they may be considered as ‘sacrificed BLUs’.

The necessary energies for the preferential sequences of propagation-bifurcation cracking, which, as previously mentioned, have been quantified for bi-material interfaces by Kendall [4] and others, can, in principle, be generalized for a multi-layer-multimaterial configuration [9]. The derivation of analytical expressions for the interfacial adhesive strength or energy of multilayered structures would provide a ranking tool for the level of resistance to fracture of the various types of cuticular interfaces. Additionally, numerical modeling would add to our understanding of interfacial failure in complex layered structures. This is currently under investigation.

### 3.2. Layer Thickness Variability in Cylindrical Structures

Among the various design strategies of structural biological materials intended to resist mechanical stresses, one type of architecture stands out, which consists of alternating concentric cylinders separated by interlayers. Examples include wood, osteonal bone, the skeletal spicules of sponges, the cuticle of some beetles, the scorpion cuticle, etc.

**Figure 8:**
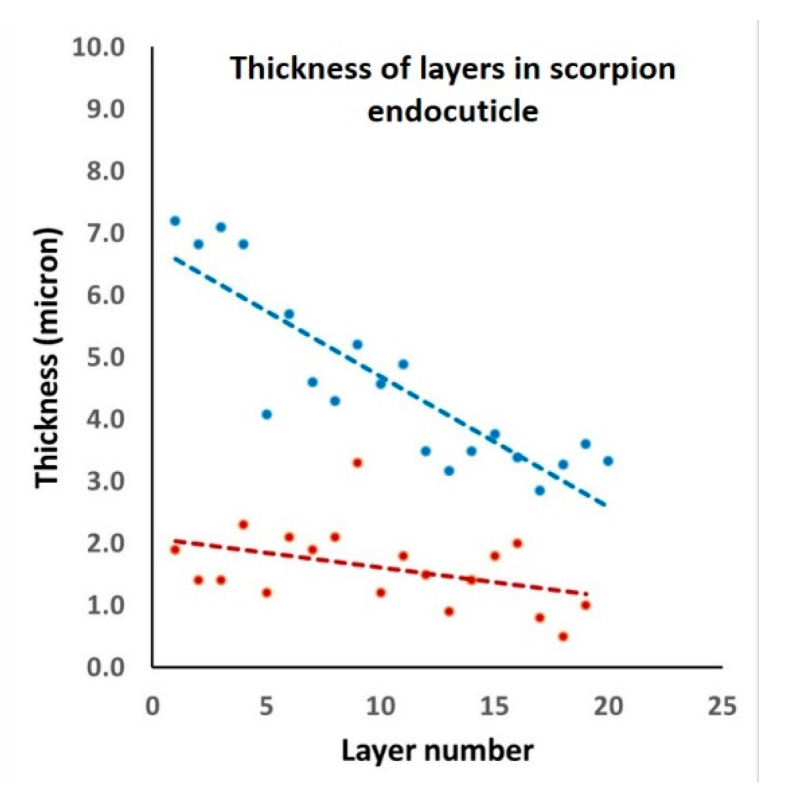
Plot of scorpion endocuticle layer thickness (blue symbols) against distance from the external side of the cylindrical cuticle (layer 1) down to its hollow core (layer 20). The much smaller interlayer thickness (red symbols, from 1 to 19) is plotted for comparison.

Focusing on the scorpion endocuticle, it can be seen from the SEM image (right-hand side of Figure 4) that, moving radially from the external side of the cylindrical cuticle down to its hollow core, the layers become increasingly thinner. From the plot in Figure 8, the layer thicknesses range from about 7 μm down to 3 μm, whereas the interlayer thickness is approximately constant, at 1–2 μm.

Whether the cylindrical layers and interlayers have specific thicknesses seems to be intimately related to the mechanical function and adaptation to the type of stresses to which the structure is subjected. The thickness of the cylindrical layers in wood or in osteonal bone are approximately equal, likely because the applied loading is mainly compression. Under bending, tension and compression stresses appear on opposite sides of the loaded structure, which then develops adaptative strategies to maximize structural strength and toughness, and reliability.

The explanation for the observed decrease in scorpion endocuticular layer thickness (Figure 8) is similar to that for the silica sponge spicule [31,32,33,34], with some important differences. According to Miserez et al. [33] and Monn et al. [34], the thickness of silica sponge layers diminishes with radial distance from the core, reaching a minimum value at the outer surface where, under bending, the stress is highest and tensile in nature. In the scorpion cuticle, however, the minimum thickness of the layers is at the core (thus the inner surface), likely because when a scorpion undergoes sharp blows or indentations from unfriendly incidents, the highest stress occurs at the core and is tensile. For layered architectures, the stress needed to cause cracks in an individual layer is proportional to t^−1/2^, where t is layer thickness [33,35]. The thinnest layer should thus have the highest strength. In addition, note that thin layers also significantly limit the depth of straight crack penetration into the structure interior [35]. The key point is that layer thickness changes appear to be a natural consequence of the increased stress applied to the scorpion cuticle and sponge spicules. Additionally, Gao et al. [36] have shown that if a characteristic dimension of a structure is smaller than a certain critical length scale (which is a function of the material and geometry of that structure), then strength no longer depends on size. In other words, at that point, nature does not need to generate thinner external layers.

Other differences between the scorpion cuticle and sponge spicules are material and structural: (i) the scorpion (endo)cuticle is a chitinous fibrous composite, whereas the spicule is a layered ceramic; (ii) the 1–2 μm thick interlayers in the scorpion endocuticle are fibrous and orthotropic, with the chitin fibers running parallel to the interface direction (they exit from the BLUs in the perpendicular direction), whereas, in the sponge spicule, those interlayers are separated by a very thin proteinic interlayer in the 5–10 nm range [35]. Both types of interlayer design contribute to a significant increase in the work of fracture and, thus, structural reliability. Indeed, a crack formed in a layer would be expected to arrest at the closest interlayer, then proceed by bifurcation or crack renucleation at a random site in a neighboring layer. SEM observations of breaks in the scorpion endocuticle (Figure 7), or of broken spicules [31,32,33,34], reveal fracture patterns consistent with such a sequential process.

Research is currently underway to derive predictive analytical and numerical approaches (for which models have begun to appear [21,37,38]) that would accurately describe the progressive fracture and increased reliability of layered natural composite structures. This could potentially be used as an inspiration for alternative designs of future engineering composites.

## Data Availability

No datasets were archived. Exact data in Figure 8 are available upon request.
